# Coupling among Microbial Communities, Biogeochemistry, and Mineralogy across Biogeochemical Facies

**DOI:** 10.1038/srep30553

**Published:** 2016-07-29

**Authors:** James C. Stegen, Allan Konopka, James P. McKinley, Chris Murray, Xueju Lin, Micah D. Miller, David W. Kennedy, Erin A. Miller, Charles T. Resch, Jim K. Fredrickson

**Affiliations:** 1 Pacific Northwest National Laboratory, Richland, WA, USA

## Abstract

Physical properties of sediments are commonly used to define subsurface lithofacies and these same physical properties influence subsurface microbial communities. This suggests an (unexploited) opportunity to use the spatial distribution of facies to predict spatial variation in biogeochemically relevant microbial attributes. Here, we characterize three biogeochemical facies—oxidized, reduced, and transition—within one lithofacies and elucidate relationships among facies features and microbial community biomass, richness, and composition. Consistent with previous observations of biogeochemical hotspots at environmental transition zones, we find elevated biomass within a biogeochemical facies that occurred at the transition between oxidized and reduced biogeochemical facies. Microbial richness—the number of microbial taxa—was lower within the reduced facies and was well-explained by a combination of pH and mineralogy. Null modeling revealed that microbial community composition was influenced by ecological selection imposed by redox state and mineralogy, possibly due to effects on nutrient availability or transport. As an illustrative case, we predict microbial biomass concentration across a three-dimensional spatial domain by coupling the spatial distribution of subsurface biogeochemical facies with biomass-facies relationships revealed here. We expect that merging such an approach with hydro-biogeochemical models will provide important constraints on simulated dynamics, thereby reducing uncertainty in model predictions.

Subsurface environments are heterogeneous three-dimensional systems characterized by variation in redox state, mineralogy, and physical structure. Spatial heterogeneity combined with limited accessibility has made it difficult to understand the coupling between subsurface physicochemical properties and microbial communities^1^. Important progress has nonetheless been made and it is clear that variation in redox state^2^,^3^, mineralogy^4^,^5^,^6^,^7^, and physical structure^8^,^9^,^10^ all impact, and are impacted by, microbial communities.

Physical and reactive chemical properties that impact microbial communities have been used to define subsurface facies^11^,^12^,^13^ due to their long-term temporal stability relative to the time-scale of microbial community dynamics. The temporal stability of facies allows characterization of their spatial distributions, and this has been leveraged in hydro-biogeochemical models^13^,^14^,^15^. This suggests that facies-microbe relationships could be coupled with facies spatial distributions to generate spatial predictions of microbial community properties.

Hydrogeological properties have previously been suggested as proxy variables to predict microbial community properties^16^. While many studies have directly or indirectly investigated microbial communities and their metabolic processes across facies^17^,^18^,^19^,^20^, the resulting microbe-facies relationships have not been leveraged to generate spatial predictions of subsurface microbial communities. In addition to generating fundamental knowledge of microbial patterns, filling this gap should provide important constraints (e.g., functional group biomass distributions) on both the initial and dynamic conditions of hydro-biogeochemical models.

We first pursue a hypothesis-driven investigation of microbe-facies relationships and then illustrate how resulting insights can be coupled with facies distributions to generate spatial predictions of microbial properties. We focus on three biogeochemical facies within a single lithofacies (Table 1 provides definitions). To enable spatial predictions we take advantage of strong vertical and weak horizontal structure in the distributions of these facies within the subsurface of the Hanford Site 300A near Richland, WA. We specifically focus on the fine-grained Ringold lithofacies^21^, which contains oxidized, reduced, and transition zones^22^,^23^. The zones identified by Lin *et al.* (2012a) and Bjornstad *et al.* (2009) have different reactive properties towards pertechnetate (TcO_4_^−^) reduction^24^ such that we refer to them as oxidized, reduced, and transition biogeochemical facies within the fine-grained Ringold lithofacies.

We evaluate the hypothesis that microbial community composition will be most strongly related to redox state—as a proxy for dominant biogeochemical processes—and that variation in sediment mineralogy, organic carbon content, and/or pH^4^,^5^,^6^,^25^,^26^,^27^,^28^ will have additional influences, but of a lesser impact. While pore structure can influence microbial communities^8^,^9^, differences are most likely to occur between facies because physical properties are relatively homogeneous within facies. To confirm this expectation, the influence of within-facies pore structure on microbial community properties was evaluated. We further hypothesize that due to mixing of complementary electron donors and acceptors, a biogeochemical ‘hot spot’ will occur in the transition biogeochemical facies, resulting in elevated microbial biomass^29^,^30^,^31^,^32^. Finally, as an illustrative case, we couple among-facies variation in microbial biomass—as revealed here—with facies spatial distributions to predict microbial biomass across a three-dimensional spatial domain.

## Methods

At the Hanford Site 300 Area, subsurface geochemical and biogeochemical processes have been identified that influence the fate and transport of contaminants^33^,^34^. A major concern at Hanford is the off-site migration of subsurface contaminants into the Columbia River^24^,^33^,^35^,^36^. Groundwater from the 300A unconfined aquifer, and a persistent uranium plume, enters the river through an extensive and dynamic groundwater-surface water interaction zone^35^. The study site is located near the inland boundary of the interaction zone and includes Plio-Pleistocene strata of the Ringold Formation—deposited by fluvio-lacustrine processes—that consists of bedded gravels, sands, and silts^37^. Those strata are overlain by unconsolidated, poorly stratified Pleistocene sands and gravels of the Hanford Formation, deposited by catastrophic floods^23^. The boundary between the Hanford and Ringold Formations is a geological unconformity where the underlying fine-grained sedimentary unit of the Ringold functions as an aquitard and contains oxidized, reduced, and transition zones^23^, here termed biogeochemical facies.

**Figure 1:**
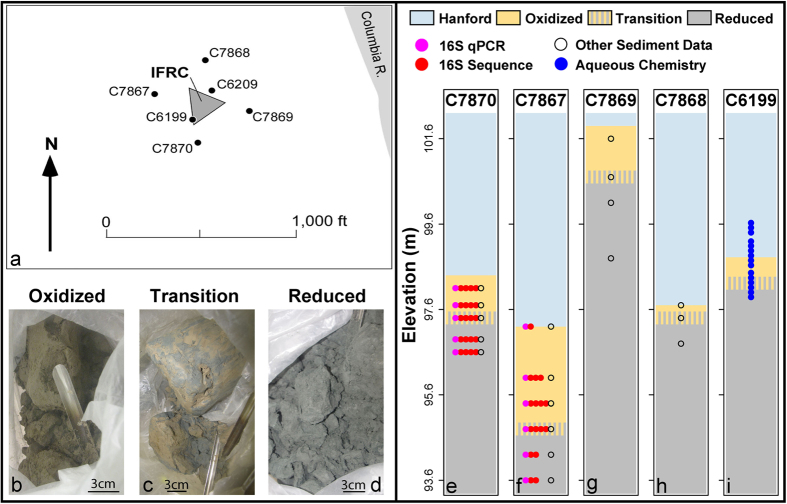
(**a**) Spatial positions of sampling locations within the Hanford Site 300 Area, adjacent to the Integrated Field Research Challenge (IFRC) site, near the Columbia River, about 2 km upstream from Richland, WA. The figure was drafted using Adobe PhotoShop CS6 Version 13.0 × 64 using well-location survey data. (**b–d**) Photos of sampled sediments from each biogeochemical facies within well C7870. (**e–i**) Vertical structure of formations, facies, samples, and number of biological replicates for each data type. Only samples used for community composition profiling via 16S rRNA gene sequencing had more than one biological replicate per depth band; the number of biological replicates that successfully sequenced is indicated by the number of solid red circles at each depth. Microbial data were collected only from C7870 and C7867. The ‘other sediment data’ category included pH, organic carbon, AVS, Fe(II), pore structure, and mineralogy. Mineralogy was not available for elevation 101.6 m in C7869 and for elevation 94.2 m in C7867 (Table 2). Pore structure data were not available for elevation 101.6 m in C7869 (Table 2).

Vertically-structured redox conditions at our field site (Fig. 1) are likely due to slow diffusion of O_2_ into the Ringold Formation from overlying aerobic groundwater within the Hanford Formation. O_2_ reacts with labile Fe(II) in the upper portion of the Ringold Formation which acts as a redox buffer against oxidation of deeper sediment. Reducing conditions are maintained beneath the upper oxidized facies by decreased O_2_ penetration and potentially by microbial consumption of O_2_ and nitrate coupled to oxidation of solid phase organic carbon^38^.

### Sediment Sampling

Sediments were collected during the sonic drilling of 7 inch diameter hydrological monitoring wells, designated C7867, C7868, C7869, and C7870 (Fig. 1). The wells surrounded the Integrated Field Research Challenge site^39^,^40^,^41^.

Sediments from the three biogeochemical facies were recovered from each borehole (Table 2); ~0.1–5 kg of core material (see Table 2) from each depth interval was collected into plastic bags in the field, and transported on wet ice to an anaerobic glove bag (Coy Laboratory Products Inc., Grass Valley MI.) that contained 95% N_2_ and 5% H_2_. Redox conditions were estimated visually based on sediment color. Between the time of sampling and placement in the anaerobic glove bag, there were no visible shifts in sediment color, indicating relatively stable redox conditions during transport. Within each depth interval of wells C7870 and C7867, we attempted to collect 4 samples ~3–5 cm from each other for microbial characterization using sterile cut-off syringes or scoops. In some cases it was not possible to collect all 4 samples and some samples failed to sequence; the number of biological replicates—and their distribution across biogeochemical facies—used in the statistical analyses is provided in Fig. 1. Each set of samples was within a single biogeochemical facies and within a distinct depth interval, but their specific spatial orientation was not controlled. Prior to subsampling in the laboratory, a fresh surface was exposed by paring away potentially contaminated sediments; samples were stored at −80 °C. Also within each depth interval of C7870 and C7867 one biological replicate was collected for DNA extraction used for qPCR of the 16S rRNA gene (see below for details). Furthermore, in all four wells at each depth interval, one biological replicate was collected for each of the following: (*i*) a sample for analysis of pore structure was collected using a cut-off 10 ml syringe to minimize disturbance, and was then vacuum impregnated in epoxy, (*ii*) a sample for acid volatile sulfide (AVS) and Fe(II) analyses was collected into a sterile 20 ml headspace vial, stoppered, weighed and flushed with N_2_, and (*iii*) additional material was collected for organic carbon, mineralogy, and pH analyses. However, mineralogy data were not available for elevation 101.6 m in C7869 and for elevation 94.2 m in C7867 (Table 2). Pore structure data were not available for elevation 101.6 m in C7869 (Table 2).

Sediment samples were categorized according to their elevation and location within the three biogeochemical facies (Fig. 1, Table 2). Elevations of the Hanford-Ringold contact and the oxidized-reduced transition facies were obtained from well logs (Table 2); vertical distances between each sample and the elevation of the redox transition were used to facilitate among-well comparisons. In some cases there was vertical fingering of oxidized and reduced sediments (e.g., Fig. 1c), and in other cases the redox boundary was visually abrupt. In both cases, samples collected immediately adjacent to a visible redox transition—brown transitioning to gray—were considered within the transition facies.

### Mineral Concentrations via X-ray Diffraction

Sediments were dried and ground using a mortar and pestle, and analyzed with an automated Bruker Topas v 4.2 (Bruker AXS, Germany) X-ray diffraction (XRD) instrument. The Rietveld method was used to reduce raw XRD spectra to phase abundances^42^. A Kruskal-Wallis test^43^ (function ‘kruskal.test’ in R package ‘stats’) and its associated post-hoc multi-comparison test^44^ (function ‘kruskalmc’ in R package ‘pgirmess’) were used to evaluate differences in mineral concentrations among biogeochemical facies. Mineral concentrations were also related to elevation using ordinary least squares regression.

### Physical Pore Structure via X-ray Tomography

For derivation of physical characteristics epoxy embedded sediment samples were imaged using a Nikon XTH 320/225 X-ray tomography system with a tungsten target at 90 kV. All acquisitions consisted of 3142 projections with each exposure lasting 1.4 seconds. The volumes were reconstructed with a voxel spacing of 16 μm. Each normalized volume was automatically segmented into clast (solid, non-epoxy) and pore space categories. All segmentations and calculations were performed using Avizo Fire v8.0^45^. Avizo documentation includes detailed descriptions of parameter derivations, which are only briefly described here. Six sample descriptors (Table S1) were calculated from the segmentations (porosity, the volumetric fraction of voids within the total volume; tortuosity, the ratio of the length of a curve to the distance between it ends; Euler index, a topological parameter describing the structure of an object; surface area per volume, ratio of the surface area to total volume; fragmentation, an index of relative surface convexity or concavity; and linear density, the number of traversals across the overall volume per unit length on a linear path). As for mineral phases, Kruskal-Wallis tests and linear regression were used to evaluate changes in each sample descriptor across facies and with elevation, respectively.

### Sulfide Inclusions

For defining the occurrence of secondary sulfide mineralization, samples from borehole C7867 at elevations 97.2 m (reduced) and 94.2 m (oxidized) were sectioned into 1 mm × 1 mm columns and imaged at the Advanced Photon Source, beamline 2-BM, with transmission collected on a scintillator-coupled CCD camera. The resultant reconstructions were examined using Avizo software to segment individual inclusions and quantify each inclusion’s sphericity, which was calculated as a function of inclusion volume and surface area. Inclusions having high sphericity and high X-ray absorption were considered to be microbially mediated precipitations of iron sulfide. Their relative abundance was calculated by comparing the percent volume they occupied in each of the two samples.

An effort was made to observe sulfide mineralization directly by constructing two-dimensional compositional maps for polished surfaces that originated within the tomographically imaged volume. The exposed surfaces were assayed using a JEOL Instruments Co., JXA-2350 electron microprobe. Of particular interest was the possible location of sulfide-rich inclusions, indicted by concentrations of sulfur at the polished surface.

### Sediment pH, Fe(II), Organic C, Acid Volatile S, and Pyritic S

Dried sediments were disaggregated with mortar and pestle prior to biogeochemical analysis. Total organic carbon was quantified with a Shimadzu SSM-5000A carbon analyzer. pH was quantified in a slurry of 1 g dry sediment mixed with 1 ml MQ water, with 10 minute equilibration prior to pH measurement. Fe(II) concentrations were measured by the ferrozine assay^46^ on extracts—1 hour equilibration—of 1 g sediment in 5 ml of 0.5 N HCl. Univariate regression was used to evaluate changes in organic carbon with changes in Fe(II) concentration, an indicator of redox state. A Kruskal-Wallis test and its associated post-hoc multi-comparison test were used to evaluate differences in organic carbon, pH, and Fe(II) among biogeochemical facies. Summary metrics are provided for acid volatile and pyritic S in the oxidized and reduced facies. Acid volatile and pyritic S were analyzed at Huffman Laboratories, Inc.(Golden, CO), according to ASTM standard methods (http://www.astm.org/DIGITAL_LIBRARY/index.shtml).

### DNA Extraction

Genomic DNA was extracted using the PowerSoil^®^-htp 96 Well Soil DNA Isolation Kit (MoBio Laboratories, Inc.) with modification to improve yield by adding casein^47^. Briefly, in each well of the 96-well plate, about 0.7 g sediment was mixed with 400 μl bead beating solution, 400 μl hot (70 °C) casein solution (containing 400 mM NaCl, 40 mM ethylenediaminetetraacetic acid (EDTA), 50 mM Tris-HCl (pH 8.0), 50 mg/ml casein, autoclaved), and 80 μl C1 solution (MoBio Laboratories, Inc.). The DNA extraction then followed the kit manual beginning from step 7. Triplicate extractions were performed for each biological replicate at each depth, and resulting DNA was pooled within each biological replicate. The DNA samples were sent to the Environmental Genomics Core Facility at the University of South Carolina to be run on a Roche FLX 454 pyrosequencing machine using the standard pyrosequencing protocols (Roche 454, Branford, CT). PCR amplification of bacterial and archaeal SSU rRNA genes was performed using the primer pairs of 515F/806R^48^.

### Processing of Sequence Data

Raw sequences were processed in QIIME^49^. Sequence reads were first binned and filtered to remove low quality sequences using default parameters. Operational taxonomic units (OTUs) were picked at 97% sequence similarity, and representative sequences were used to identify chimeric sequences using ChimeraSlayer^50^. Taxonomy was assigned to each representative sequence using BLAST against the Greengenes core set^51^. For all OTU-based analyses, the original OTU table was rarefied to a depth of 1000 sequences per sample, to minimize the effects of different sampling effort in comparing beta-diversity across the samples. The number of sequences attributed to a given OTU within a given community was divided by 1000 to provide an estimate of that OTU’s relative abundance within the community.

### Abundance Estimates

To estimate abundances of the bacterial 16S rRNA gene, qPCR assays were performed on DNA extracts following Lin *et al.*^22^. All qPCR assays were performed with a StepOnePlus Real Time PCR system (Applied Biosystems Inc., Foster City, CA) programmed for 40 cycles according to Lin *et al.*^22^. A subset of DNA extractions—one biological replicate per depth—were used for qPCR assays to span the three biogeochemical facies, and within each three technical replicates were run. This enabled visualization of clear shifts in microbial abundance across biogeochemical facies, but insufficient data to perform formal statistical tests.

### Microbial Community Analysis

To study how microbial OTU richness varied across communities, forward model selection—based on the small sample size Akaike Information Criterion^52^,^53^ —was used to generate a parsimonious multiple-regression model with the following as potential explanatory variables: mineral concentrations, tomography-based physical descriptors, Fe(II), elevation relative to the redox transition, microbial abundance, and organic carbon concentration. In addition, a Kruskal-Wallis^43^ test was used to evaluate shifts in OTU richness across biogeochemical facies.

To determine whether microbial community composition was associated with biogeochemical facies we first visualized compositional relationships using nonmetric multidimensional scaling^54^ (NMDS) based on Bray-Curtis^55^ dissimilarities (R function ‘metaMDS’ in package ‘vegan’). To determine whether microbial community composition was influenced by environmental conditions beyond redox conditions, a dissimilarity matrix was computed using the β-nearest taxon index (βNTI) as described in Stegen *et al.*^56^,^57^ using R (http://cran.r-project.org/). The βNTI metric was used because—relative to Bray-Curtis—it more rigorously identifies environmental variables that cause differential fitness among microbial taxa and, in turn, influence community composition. Mineral abundances, values for structural (i.e., tomographic) descriptors, organic carbon concentrations, bacterial abundance estimates, Fe(II) concentrations, pH, and elevation relative to the redox transition were used to calculate magnitudes of change in each of those variables between each sample. Between-sample differences in each variable were, in turn, used to generate variable-specific dissimilarity matrices.

Forward model selection was used to determine which environmental variables best explained βNTI. Because βNTI is a distance metric these analyses relate the βNTI distance matrix against distance matrices of explanatory variables. Data are non-independent within a distance matrix, and to appropriately account for this non-independence we used Mantel^58^ and partial Mantel^59^ tests (R function ‘mantel’ in package ‘ecodist’) using 1000 permutations to evaluate significance at each step in the model selection. Variables with the strongest correlation (or partial correlation) with βNTI were added at each step in the model selection. Model selection was terminated when the next variable to be added was not significantly related to βNTI, as evaluated by a partial Mantel test.

### Groundwater Sampling and Analysis

Changes in geochemistry with depth and across stratigraphic boundaries were determined from groundwater samples collected at 0.1 m intervals using multi-level sampler methods^60^ in well C6199 (Fig. 1). Sampling in Well C6199 (Fig. 1) spanned all three biogeochemical facies.

At each sampled interval a chemically inert cell—capped by 0.45 μm filter membranes—was filled with deionized water and suspended on a support rod within the well screen. The support rod included centralizing disks to stabilize the sample assemblage, and vertically-arrayed cells were separated by flexible baffles that registered with the well screen. To sample dissolved gases, gas-tight syringes were attached to the support rods with gas-permeable tubing affixed to the syringe opening. Groundwater equilibrated with the syringes and cells over a deployment of ~500 h.

Anion concentrations were measured using a Dionex ICS-2000 anion chromatograph with an IonPac AS18 (4 × 250 mm) column (Dionex. Inc.), 22 mM KOH eluent at 1 mL/min isocratic for 15 minutes at 30 °C and a 57 mA anion suppressor current. Standards were made from Spex CertiPrep, Metuchen, NJ, 08840; anion standards were calibrated from 0.24 to 120 ppm (except fluoride, 0.08 to 40 ppm).

Cation concentrations were determined on nitric acid-acidified samples with a Perkin Elmer Optima 2100 DV ICP-OES. A Helix Tracey 4300 DV spray chamber and SeaSpray nebulizer were used with double distilled 2% nitric acid (GFS Chemicals, Inc. Cat. 621) at 1.5 mL/min. Calibration standards were made with Ultra Scientific ICP standards (Kingstown, RI), from 0.5 to 3000 μg/L.

## Results

### Sediment Mineralogy and Geochemistry

**Figure 2:**
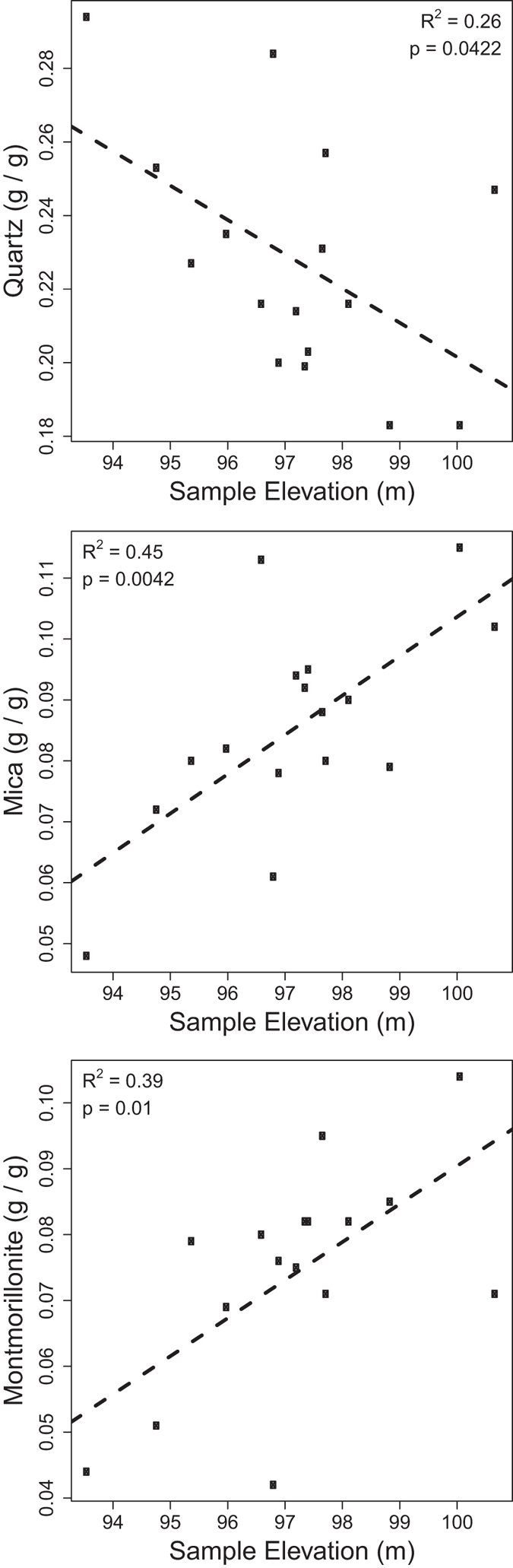
Mineral concentrations related to elevation. Linear regression statistics are provided on each panel and dashed lines indicate the regression model; only minerals that were significantly related to elevation are shown.

Rietveld Refinement estimated mineral abundances with small differences between observed and calculated intensities (Fig. S1). The sediments consisted of common rock-forming minerals and amorphous phases (Table S2). Clinochlore concentrations were higher in the oxidized facies than in the reduced facies (p < 0.05, by Kruskal-Wallis), but concentration in the transition facies was not different from the other two (p > 0.05). No other mineral showed significant differences among facies (p > 0.05, for all). Linear regression revealed that quartz decreased significantly with elevation (p = 0.04, R^2^ = 0.26), and mica (p = 0.004, R^2^ = 0.45) and montmorillonite (p = 0.01, R^2^ = 0.39) increased significantly with elevation (Fig. 2); all other mineral phases did not vary significantly with elevation (p > 0.05, for all).

**Figure 3:**
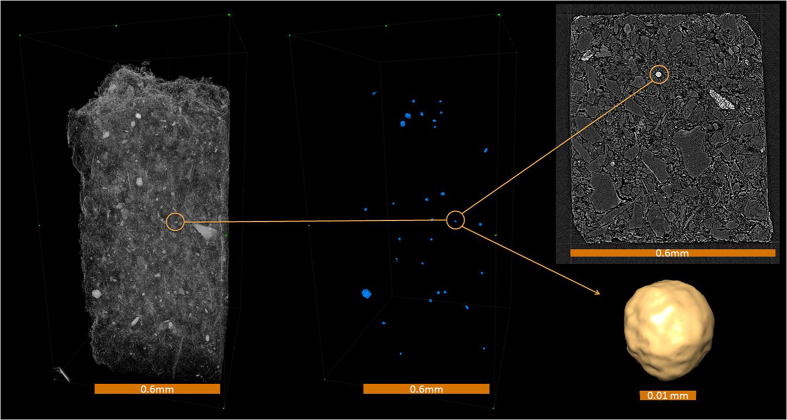
CT reconstructions showing (Left) 3D volume reconstruction of reduced biogeochemical facies sediment; (Middle) isolated occurrences of biogenic iron sulfide minerals; (Right) a single-voxel slice reconstruction and its included iron sulfide framboid. The position of that framboid in each representation is indicated.

Compositional maps revealed iron sulfide framboids (Fig. 3) consistent with previous TEM and SEM of material from the same reduced facies collected from a nearby borehole^24^. In the reduced facies sample from this study, 0.47 mm^3^ of sediment contained 70 spheres occupying a total volume of 8.2 × 10^−5^ mm^3^, which represented 0.0175% of the sample volume; in the oxidized facies sample 1.22 mm^3^ of sediment contained 3 spheres, occupying a total volume of 1.05 × 10^−5^ mm^3^, which represented 0.0009% of the sample volume. The reduced facies therefore contained ~20 times the volume of iron sulfide framboids than the oxidized facies. Similarly, pyritic S ranged from 9–37μmol/g in the reduced facies, but was below detection in the oxidized facies, also consistent with previous ^57^Fe Mössbauer spectroscopy analyses of the oxidized and reduced fine-grained Ringold sediment^24^.

Physical sample descriptors estimated from X-ray tomography (e.g., porosity, etc.) did not vary across biogeochemical facies (p > 0.05, for all by Kruskal-Wallis) and did not vary significantly with elevation (p > 0.05, for all by linear regression). A Kruskal-Wallis test and its associated post-hoc test revealed that pH and Fe(II) concentration were significantly higher in the reduced than in the oxidized facies (p < 0.05; Fig. S2); pH and Fe(II) in the transition facies did not differ significantly from the other two facies (p > 0.05; Fig. S2). Organic carbon concentration in Ringold sediments did not vary significantly across biogeochemical facies (p > 0.05, by Kruskal-Wallis) or with Fe(II) concentration (p > 0.05, by linear regression); the relationship with Fe(II) remained non-significant when two extreme outliers were removed.

### Microbial Communities

**Figure 4:**
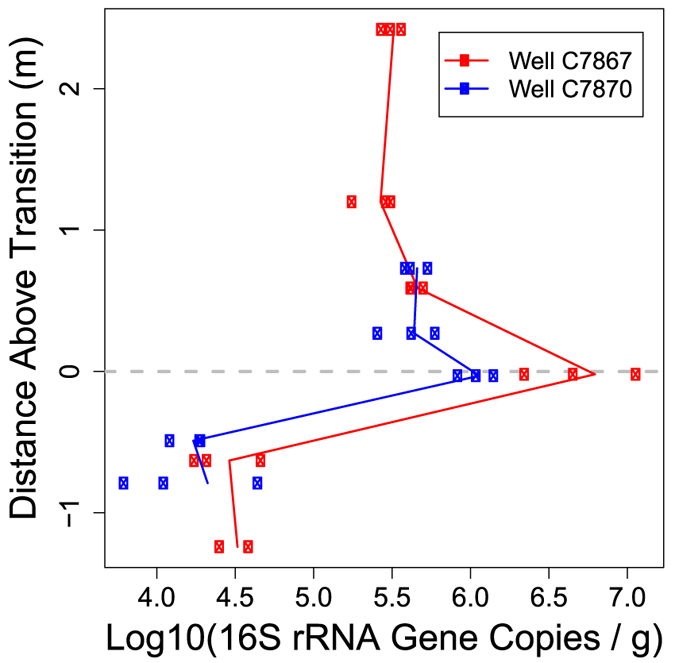
qPCR-based estimates of 16S rRNA gene copies per gram of sediment—as a proxy of microbial biomass—in two wells, as a function of the vertical distance above the transition biogeochemical facies. Multiple points at each elevation represent technical replicates. Solid lines connect mean gene abundances within each well.

**Figure 5:**
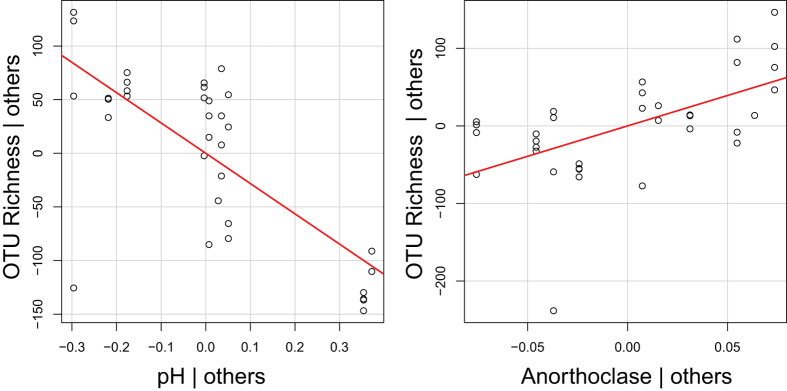
Partial regression plots showing contribution of each variable retained in the OTU richness multiple-regression model, holding the other retained variable constant. Both retained variables were significant (p ≪ 0.05) and the model R^2^ was 0.65.

Microbial abundance—using qPCR of the 16S rRNA gene as a proxy—was highest in the redox transition facies for both wells, and was on average 10–20 fold higher than in the reduced facies (Fig. 4). OTU richness was well-explained by a two-variable multiple-regression model (R^2^ = 0.65; p ≪ 0.0001) whereby richness declined with increasing pH and increased with increasing anorthoclase abundance (Fig. 5). OTU richness was significantly lower in the reduced facies than in the other two facies (p < 0.05, by Kruskal-Wallis; Fig. S3). Futhermore, a total of 2618 unique OTUs were observed across all communities. There were 1756, 1013, and 784 OTUs found in the oxidized, transition, and reduced facies. Of these, 1054, 436, and 388 were only found in the oxidized, transition, and reduced facies. The oxidized and transition facies shared 539 OTUs, the oxidized and reduced facies shared 358 OTUs, the transition and reduced facies shared 233 OTUs, and 195 OTUs were found across all three facies.

**Figure 6:**
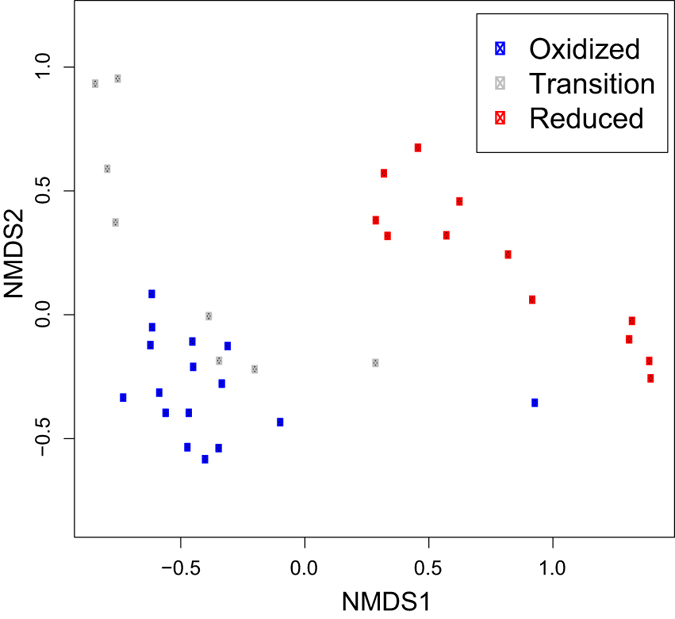
NMDS plot showing clustering of microbial communities based on Bray-Curtis dissimilarity.

**Figure 7:**
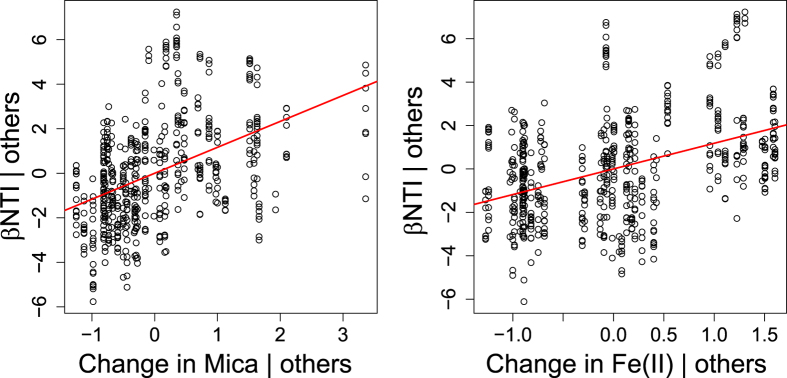
Partial regression plots showing contribution of both variables retained within the βNTI multiple regression model, holding the other retained variable constant.

With respect to overall community composition, communities clustered based on biogeochemical facies (Fig. 6). Model selection for βNTI revealed a two-variable model whereby βNTI increased with increasing shifts in mica and Fe(II) concentrations (p = 0.001 for both variables; Fig. 7). In addition, the relative abundances of 26 OTUs were significantly correlated with Fe(II) concentrations, and 24 of these were negative relationships (Fig. S4). The taxonomic affiliations of these 26 OTUs were examined, but no clear patterns emerged (Fig. S4).

### Groundwater

The concentration of 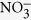 in MLS samples decreased with increasing depth across the redox transition with a concurrent increase in 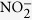 followed by a decrease to below detection. Soluble Fe and Mn were below detection except near the bottom of the screened interval where they increased to 144 and 511 μg L^−1^, respectively, concurrent with the decrease of nitrate and nitrite to below detection (Fig. S5). S^2−^ was not detected in any of the MLS samples examined as part of this investigation but has been detected just below the redox interface^22^, suggesting this analyte is spatially and/or temporally variable.

## Discussion

### Geochemistry and Mineralogy

Sediment samples examined here originated below the geologic unconformity that forms the interface between the top of the Ringold mud lithofacies and the base of the Hanford Formation. The Ringold mud lithofacies is representative of a fluvial overbank (low energy) depositional environment whereas the Hanford is coarse-grained and was deposited during high-energy Pleistocene floods^23^. The unconfined aquifer within the Hanford Formation is oxic and contains three primary lithofacies: gravel-dominated, sand-dominated, and interbedded sand- and silt-dominated^23^.

While our sediment samples focused on biogeochemical facies within the Ringold mud lithofacies, our aqueous geochemical profile spanned Hanford and Ringold lithofacies and clearly showed a transition to increasingly reduced conditions below the unconformity. Concomitant with the vertically structured shift in reducing conditions, the abundances of three minerals—quartz, mica, and montmorillonite—varied significantly with subsurface elevation in the Ringold mud lithofacies; quartz declined while mica and montmorillonite increased towards higher elevation. This can result from decreasing energy during deposition, whereby larger particles (quartz) deposit at the base and fines (mica and montmorillonite) become more abundant towards the top. This fining-upward sedimentary sequence is common within the Ringold Formation^21^ and likely resulted from overbank or lacustrine deposition.

Surprisingly, clinochlore was the only mineral phase that varied significantly across biogeochemical facies, and was higher in the reduced than in the oxidized facies. Between-well differences in the elevations at which biogeochemical facies were encountered (Table 2) allowed us to distinguish between elevation-associated and facies-associated mineralogical patterns. The resulting patterns suggest that spatial variation in some mineral phases may be associated with depositional processes, others (i.e., clinochlore) with redox conditions, and still others appear to be unrelated to deposition or redox. In contrast, properties determined by tomography (Table S1) did not vary significantly with elevation or across biogeochemical facies, confirming that sediment sampling occurred within a single lithofacies (i.e., the fine-grained Ringold).

The deposition of gravels within the Hanford lithofacies during the Missoula Floods occurred on an erosional surface that included the top of the fine-grained Ringold lithofacies in the study area. The presence of dissolved O_2_ in groundwater associated with the Hanford lithofacies may have promoted the oxidation of organic carbon and inorganic species (e.g., Fe^2+^, S^2−^) by diffusing into the underlying Ringold sediments. Assuming a homogeneous distribution of organic carbon this scenario should lead to a positive relationship between organic carbon concentration and Fe(II), but we did not observe such a relationship. Instead, the organic carbon data contained extreme outliers that suggested a heterogeneous distribution as has also been observed in subsurface sediments associated with the eastern U.S. Atlantic coastal plain^25^.

During coring operations isolated pockets of woody fragments were observed within the Ringold mud lithofacies. As noted above, the fining upward sequence indicated by vertical variations in mineral content suggests minimal disturbance to sampled Ringold sediments, which implies that these wood fragments were buried during deposition. In this case, it is not surprising to find heterogeneously distributed pockets of high organic carbon concentrations.

We speculate that heterogeneously distributed organic carbon deposits also drive localized hot-spots of biogeochemical activity due to the same mechanisms that resulted in the hot-spot of microbial biomass observed within the transition biogeochemical facies (Fig. 4). That is, the reduced zone is likely limited by electron acceptor availability whereas the oxidized zone is electron donor limited^38^. Biogeochemical hot spots in the subsurface likely occur at interfaces where these two conditions overlap^30^, as suggested by high microbial biomass in the transition biogeochemical facies (Fig. 4, discussed below). Localized organic carbon deposits occurring within the oxidized zone would similarly provide electron donor within a broader matrix that provides high-energy yielding terminal electron acceptors such as O_2_ and NO_3_. At the interface between oxidized sediments and a localized organic carbon deposit we would, therefore, expect high microbial biomass and metabolic activity, and thus a relatively localized hot spot. While batch microcosm experiments performed with disaggregated sediments can be used to explore physical, chemical, and biological factors affecting biogeochemical rates^61^, time-course sediment incubations performed in the laboratory can greatly overestimate *in situ* rates^62^ and therefore were not performed here.

### Microbial Community Biomass, Richness, and Composition

Microbial community biomass was clearly elevated at the redox transition, which is consistent with the hypothesis that microbial activity is stimulated in biogeochemical transition zones^30^. Our working hypothesis is that the juxtaposition—within the transition biogeochemical facies—of complementary electron donors (from the reduced facies) and terminal electron acceptors (from the oxidized facies) supports higher microbial biomass, which may elevate biogeochemical rates^16^,^63^. Electron donors could result from microbial fermentation of sediment-associated organic carbon in the reduced facies^25^,^32^. Sediment-associated organic carbon was not elevated within the reduced facies, however, suggesting the need to evaluate organic carbon bioavailability and composition across biogeochemical facies.

The number of unique OTUs observed within communities was lowest in the reduced facies and was best explained by a combination of pH and anorthoclase abundance. Within the associated multiple-regression model, OTU richness declined with increasing pH and increased with increasing anorthoclase concentration (Fig. 5). The decline in OTU richness with increasing pH is consistent with observations in soil systems, where OTU richness begins to decline across the sampled pH range^27^. The increase in OTU richness with increasing anorthoclase is more difficult to interpret. Anorthoclase was not significantly related to elevation, suggesting that it cannot be interpreted as a proxy for the depositional environment. Anorthoclase may instead be associated with bioavailability of nutrients as in Rogers and Bennett^64^, where weathering of P-containing mineral inclusions stimulated growth of microbial biomass; measurements of bio-available nutrient inclusions were not, however, available in the present study. Depressed OTU richness in the reduced facies is in contrast to patterns from marine sediments, where bacterial richness was highest in reduced sediments^65^,^66^. This suggests that redox-state itself does not govern microbial richness in the subsurface; instead, microbial richness appears to be governed more by pH and mineralogy. As noted above, however, particle size distributions were not determined for the different mineral classes and X-ray tomography was unable to resolve pore structure below 16 μm. We cannot, therefore, exclude the possibility that the relationship between mineralogy and microbial richness was indirect, with richness being governed more directly by mineral particle size distributions and their impact on physical properties. The lack of a relationship between OTU richness and organic carbon concentration is also intriguing as it would seem to deviate from macro-ecological observations in which taxonomic richness increases with energy supply^67^,^68^,^69^. This again highlights the need to evaluate organic carbon bioavailability and chemical characteristics across the biogeochemical facies.

Microbial community composition was clearly differentiated between the oxidized and reduced biogeochemical facies—as indicated by both the NMDS and βNTI analyses—but a distinctive community did not emerge within the transition biogeochemical facies (Fig. 6). In addition, with increasing change in Fe(II), βNTI trended towards values greater than +2, which is the threshold above which one can infer that a change in environmental conditions causes a change in community composition due to the environment selecting for some taxa and against others^57^,^70^,^71^. This result indicates that changes in the identity of prevailing terminal electron acceptors drive shifts in microbial composition via deterministic ecological selection. βNTI was, however, also strongly related to mica concentration indicating that mineralogy and redox state jointly influence community composition. Previous work has shown that microbial community composition is impacted by mineral composition, whereby microbes apparently scavenge trace nutrients from mineral phases^4^,^5^,^6^,^64^. Different minerals in soil have also been shown to select for distinct microbial communities^4^, possibly due to the release of specific elements as the minerals weather. Due to lack of information on the elemental composition of mica in our samples, however, we are unable to relate specific elements to microbial community composition.

### Predicting Microbial Community Properties Using Facies Distributions

Placing microbial ecology in the context of subsurface facies has revealed that microbial community properties—biomass, richness, composition—are related to (*i*) properties that vary systematically across biogeochemical facies (e.g., redox state) and (*ii*) additional properties that do not vary systematically across biogeochemical facies (e.g., mineralogy). Facies are relatively stable in time and can thus be mapped in three-dimensions with some assurance that the mapping will remain valid for a significant period of time. Microbial community properties that are strongly related to facies properties can, in turn, be similarly mapped; this is one way to use ‘proxy’ variables^16^ to predict spatial distributions of subsurface microbial properties. Spatial characterization of sulfate reducing bacterial (SRB) activity, for example, revealed a strong influence of porosity^10^; in principle, spatially-explicit characterization of porosity could therefore be used to generate spatial projections of SRB activity as a model input. This approach has received limited attention despite its potential to improve predictions of hydro-biogeochemical models.

**Figure 8:**
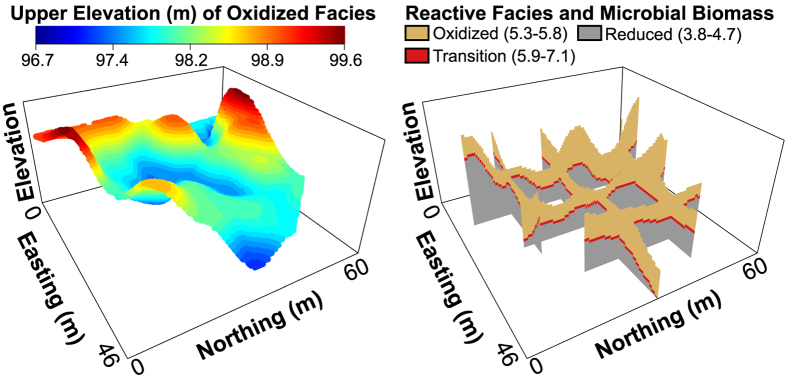
(Left) Spatial variation in the upper elevation of the oxidized biogeochemical facies across the IFRC field site and well C6209 (see Fig. 1). (Right) Spatial distribution of biogeochemical facies and predicted spatial variation in 16S rRNA gene copy abundance—a proxy for microbial biomass; facies-specific ranges in gene copy number (copies/g) are provided in Log_10_ transformed units. The vertical elevation axis in both panels ranges from 96–100 m.

As an illustrative example of this approach, we generated a three-dimensional map of microbial biomass based on the profile of biomass across biogeochemical facies (Fig. 4) and the spatial distributions of biogeochemical facies in the IFRC (Fig. 1). To do so we used geologist well logs to define elevations of the redox transition and the Hanford-Ringold contact—this is the upper elevation of the oxidized biogeochemical facies—for each of the IFRC’s 35 boreholes^23^. Those elevations were spatially interpolated to generate a surface for each transition; combining those two surfaces enabled a three-dimensional map of biogeochemical facies and therefore a map of microbial biomass (Fig. 8) that can mediate important functions such as organic carbon oxidation and maintenance of redox conditions^38^. For modeling purposes, the biomass predictions shown in Fig. 8 could be made more realistic by stochastically assigning biomass values to grid cells based on the distribution of biomass estimates within each biogeochemical facies. Alternatively, if spatially-structured features were identified as influencing microbial biomass within each facies, these features could be coupled with facies spatial distributions to predict microbial biomass across and within facies. While we focus on biogeochemical facies, this approach could also be applied across lithofacies to enable broader predictions of microbial community properties. Doing so would facilitate the application of multi-scale models to work towards increasingly robust field-scale predictions of biogeochemical function.

## Additional Information

**How to cite this article**: Stegen, J. C. *et al.* Coupling among Microbial Communities, Biogeochemistry, and Mineralogy across Biogeochemical Facies. *Sci. Rep.* **6**, 30553; doi: 10.1038/srep30553 (2016).

## Supplementary Material

Supplementary Information

## Figures and Tables

**Table 1:** Definitions of key terms; some definitions are specific to their use in this study.

Term	Definition
Aquitard	Low permeability material that prevents or greatly retards water movement between or within an aquifer.
Biogeochemical facies	A geological unit with specific biogeochemical properties such as redox chemistry or reactivity.
Bray-Curtis	Multivariate statistic that uses OTU relative abundances to quantify the difference in community composition between a pair of communities.
Ecological selection	Selection is the result of biotic and abiotic pressures causing variation in reproductive success across individuals and species.
Fermentation	Energy yielding reaction in which organic compounds are the primary electron donor and the ultimate electron acceptor.
Fluvio-lacustrine	Sediments deposited by ancient rivers or lakes.
Framboid	Spherical micromorphology of pyrite (FeS_2_) common to some anoxic sediments.
Lithofacies	A geologic unit with specific petrological characteristics such as grain size and mineralogy.
β-mean nearest taxon distance (βMNTD)	Multivariate statistic that (*i*) uses the phylogenetic distance between each OTU in one community and its closest relative in a second community to (*ii*) quantify the average phylogenetic distance between two communities.
β-nearest taxon index (βNTI)	Quantifies the difference between observed βMNTD and the mean of the βMNTD null distribution, in units of standard deviations.
Nonmetric multidimensional scaling (NMDS)	Statistical method used to collapse high-dimensional data onto a small number of axes to facilitate interpretation and visualization.
Null model	Randomization used here to generate βMNTD values expected if community assembly was not influenced by ecological selection; repeating the randomization many times provides a distribution of null βMNTD values, used in the calculation of βNTI.
Operational Taxonomic Unit (OTU)	16S rRNA gene sequences similar enough to each other—based on a pre-defined threshold—to be grouped together prior to statistical analysis.
Redox	Contraction of oxidation-reduction; net oxidation state of sediment based on the oxidation state of individual elements (i.e., Mn, Fe, S).
OTU richness	The number of unique OTUs present in a sample or community.
Terminal electron acceptor (TEA)	Oxidized form of an element used by microorganisms for the biochemical oxidation of organic carbon or reduced inorganic compounds (electron donors) to generate energy.
Transition zone	Spatial domains where properties of interest (e.g., redox) change dramatically over distances much shorter than the scale of the whole system.

**Table 2:** Characteristics of sampled wells used for sediment sampling and sample elevations and redox conditions.

Well		Elev. (m)	Facies	Microb.	XRD	Tomo.	Mass (kg)
C7870							
SE:	115.2	98.1	O	X	X	X	1
HRE:	98.4	97.7	O	X	X	X	4
TE:	97.4	97.4	T	X	X	X	4
		96.9	R	X	X	X	1
		96.6	R	X	X	X	4
C7869							
SE:	114.7	101.6	O				0.1
HRE:	101.9	100.7	T		X	X	3
TE:	100.7	100.1	R		X	X	5
		98.8	R		X	X	0.6
C7868							
SE:	114.8	97.7	O		X	X	0.7
HRE:	97.7	97.4	T		X	X	0.6
TE:	97.4	96.8	R		X	X	0.8
C7867							
SE:	115.2	97.2	O	X	X	X	1.3
HRE:	97.2	96.0	O	X	X	X	1.4
TE:	94.8	95.4	O	X	X	X	1.4
		94.8	T	X	X	X	1.5
		94.2	R	X		X	1.8
		93.6	R	X	X	X	1.3

Not all samples are associated with all data types; organic

carbon, pH, and Fe(II) were sampled at all locations; an ‘X’ in the Microb., XRD, and Tomo.

columns indicate, respectively, that estimates exist for microbial community composition

and biomass, mineralogy, and tomographic structure. SE = well surface elevation, HRE =

elevation within the well of the Hanford-Ringold interface, TE = elevation within the well of

the transition biogeochemical facies, O = oxidized facies, R = reduced facies, I = transition facies.

Approximate wet masses of collected sediments at each depth are also provided.
