# Supplementary material for: Coupling among Microbial Communities, Biogeochemistry, and Mineralogy across Biogeochemical Facies
Source: Sci Rep. 2016 Jul 29;6:30553. doi: 10.1038/srep30553 (PMC4965824; doi:10.1038/srep30553)

Coupling among Microbial Communities, Biogeochemistry, and Mineralogy across Biogeochemical Facies

James C. Stegen[1,*], Allan Konopka[1], James P. McKinley[1], Chris Murray[1], Xueju Lin[1], Micah D. Miller[1], David W. Kennedy[1], Erin A. Miller[1], Charles T. Resch[1], and Jim K. Fredrickson[1]

[1]Pacific Northwest National Laboratory, Richland, WA, USA

*Corresponding Author: James Stegen, Phone (509) 371-6763; Fax (509) 371-6955

E-mail: james.stegen@pnnl.gov

Supplemental Tables and Figures

Table S1. Physical pore structure as determined by X-ray tomography across wells and elevations (El., m). RF = reactive facies, Norm. Euler = normalized Euler, Surf. Area = surface area per volume, Frag. Index = fragmentation index, Lin. Den. = linear density.

Well	El., m	RF	Porosity	Tortuosity	Norm. Euler	Surf. Area	Frag. Index	Lin. Den.
C7870	98.1	O	0.316	4.385	0.000504	1.09E-05	11.699	0.476
C7870	97.7	O	0.053	8.565	0.000409	3.29E-06	19.101	0.144
C7870	97.4	T	0.036	20.816	1.26E-05	2.01E-07	21.54	0.009
C7870	96.9	R	0.112	10.154	0.000229	3.62E-06	15.051	0.159
C7870	96.6	R	0.204	6.312	0.000299	4.23E-06	17.202	0.186
C7869	100.7	T	0.159	3.108	0.00097	1.91E-05	6.077	0.836
C7869	100.1	R	0.065	8.34	0.000154	4.22E-06	17.984	0.185
C7869	98.8	R	0.1	4.798	5.48E-05	3.11E-06	15.031	0.137
C7868	97.7	O	0.028	7.556	0.00105	1.23E-05	12.867	0.541
C7868	97.4	T	0.332	3.639	0.000248	8.76E-06	15.447	0.372
C7868	96.8	R	0.266	2.578	6.74E-05	4.24E-06	13.181	0.186
C7867	97.2	O	0.207	6.447	0.000167	3.81E-06	16.012	0.167
C7867	96.0	O	0.021	9.336	0.000569	5.43E-06	20.064	0.238
C7867	95.4	O	0.135	6.759	0.000331	5.41E-06	17.92	0.237
C7867	94.8	T	0.08	8.986	0.000141	1.92E-06	23.6	0.084
C7867	94.2	R	0.03	22.612	0.000127	1.29E-06	22.222	0.056
C7867	93.6	R	0.09	4.794	0.000569	1.19E-05	12.134	0.521

Table S2. Mineralogy—as percent by volume—of Ringold sediments across wells and elevations (El., m) as determined by Rietveld refinement of X-ray diffraction results. RF = reactive facies, Qtz =quartz, Ab =albite, Ancl =anorthoclase, Mont = montmorillonite, Heul = heulandite, Clcl = chlinochlore, Hbd = hornblende, Amor = amorphous.

Well	El., m	RF	Qtz	Ab	Ancl	Mica	Mont	Heul	Clcl	Hbd	Amor
C7870	98.1	O	21.6	18.0	11.1	9.0	8.2	2.7	2.4	1.0	26.0
C7870	97.7	O	23.1	12.5	9.7	8.8	9.5	4.6	3.5	0.7	27.5
C7870	97.4	T	19.9	13.5	14.4	9.2	8.2	3.0	3.8	0.0	28.0
C7870	96.9	R	20.0	17.2	5.9	7.8	7.6	3.0	5.2	0.5	32.9
C7870	96.6	R	21.6	11.4	9.7	11.3	8.0	3.0	5.1	0.9	28.9
C7869	100.7	T	24.7	11.6	14.2	10.2	7.1	12.2	3.7	2.0	14.4
C7869	100.1	R	18.3	13.1	11.4	11.5	10.4	5.9	4.4	1.3	23.7
C7869	98.8	R	18.3	11.0	10.5	7.9	8.5	6.0	4.5	0.5	32.9
C7868	97.7	O	25.7	9.7	13.7	8.0	7.1	3.1	3.3	1.1	28.2
C7868	97.4	T	20.3	12.0	13.4	9.5	8.2	4.4	3.1	0.5	28.6
C7868	96.8	R	28.4	14.8	14.2	6.1	4.2	7.8	4.9	1.8	17.8
C7867	97.2	O	21.4	7.8	20.1	9.4	7.5	8.1	2.9	1.8	20.9
C7867	96.0	O	23.5	20.0	17.8	8.2	6.9	7.0	2.5	0.9	13.4
C7867	95.4	O	22.7	19.3	21.1	8.0	7.9	6.7	3.5	1.1	9.8
C7867	94.8	T	25.3	11.0	19.1	7.2	5.1	7.0	3.8	0.2	21.5
C7867	93.6	R	29.4	14.2	13.7	4.8	4.4	3.2	2.1	1.3	27.0

Figure S1. Representative example of Rietveld refinement of X-ray diffraction data for Ringold sediment sample. Data shown are from Well C7867 at elevation 96.0 m. Peaks attributed as follows: mon = montmorillonite, mic = mica, heu = heulandite, hor = hornblende, f = feldspar, q = quartz, z = zincite (internal standard).

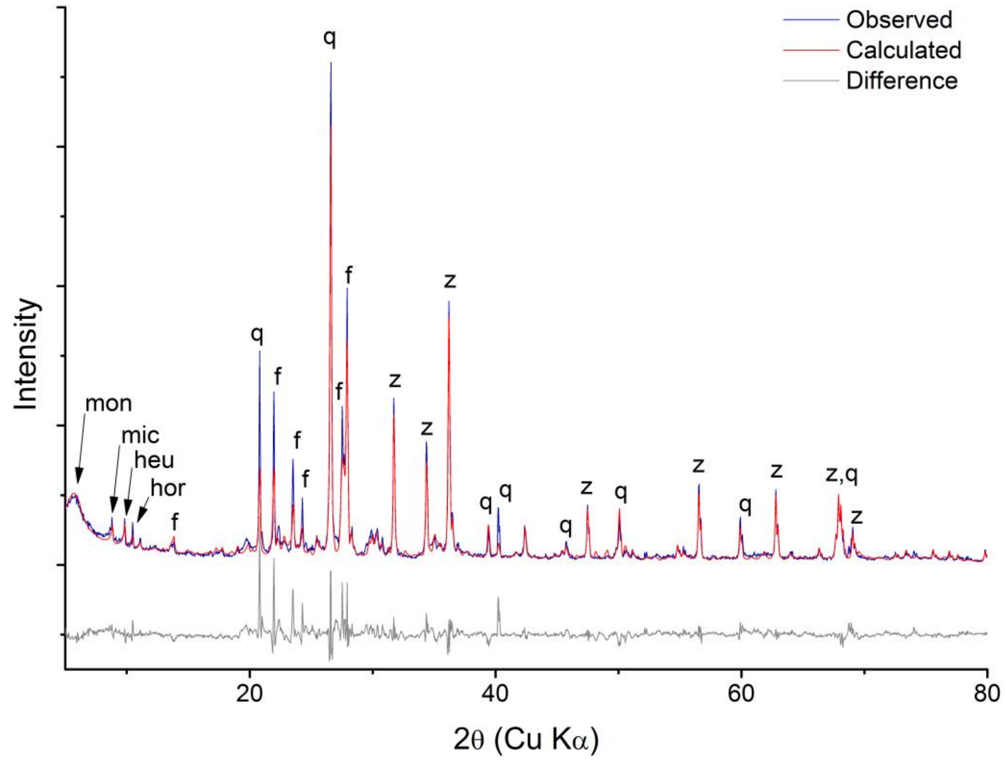

Figure S2. Boxplots of Fe(II) concentration and pH across reactive facies; letters designate significant differences (or not) between each facies, as evaluated by the post-hoc complement of a Kruskal-Wallis test.

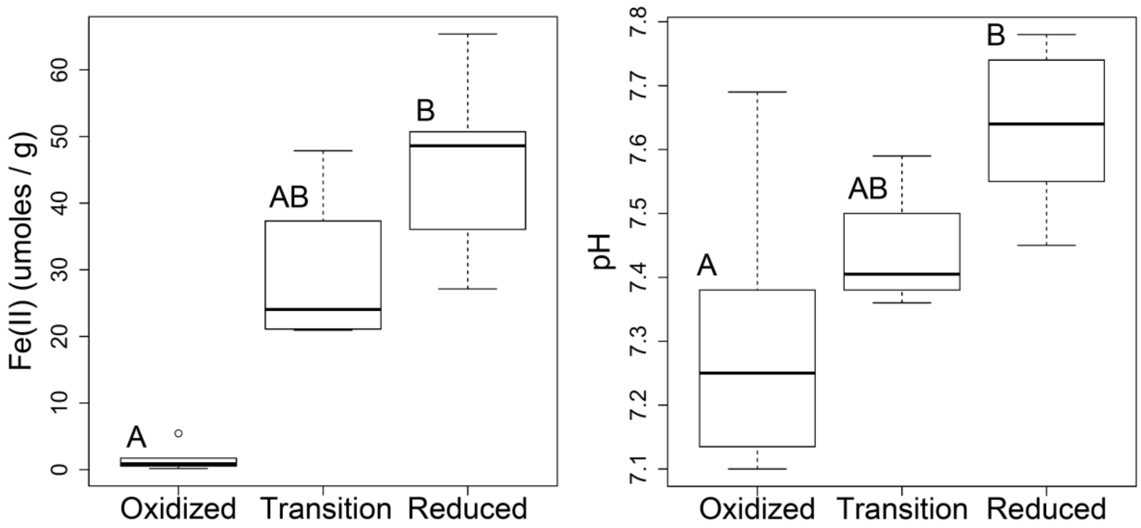

Figure S3. Boxplots of OTU richness across reactive facies; letters designate significant differences (or not) between each facies, as evaluated by the post-hoc complement of a Kruskal-Wallis test.

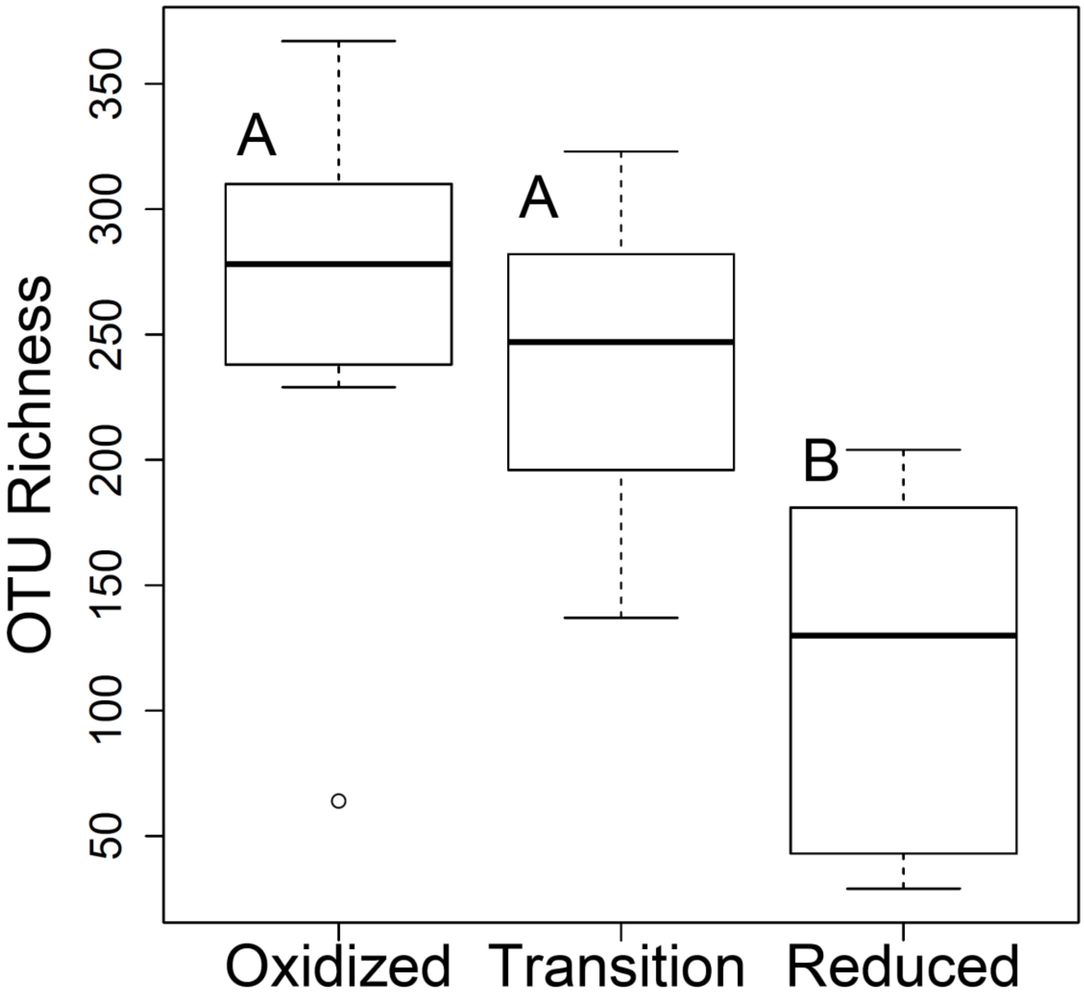

Figure S4. Significant relationships between OTU relative abundance and Fe(II) concentration. The Spearman Rank correlation was used due to non-linearity; 2545 OTUs were evaluated, so Bonferroni correction was used to evaluate significance; the corrected significance threshold was 0.05 / 2545 = 1.96 x 10^{-5}. Correlation statistics are provided on each panel. The phylum ("p__") and highest assigned taxonomic resolution are provided; "c__" = class, "f__" = family, "g__" = genus. No species-level identifications were made.

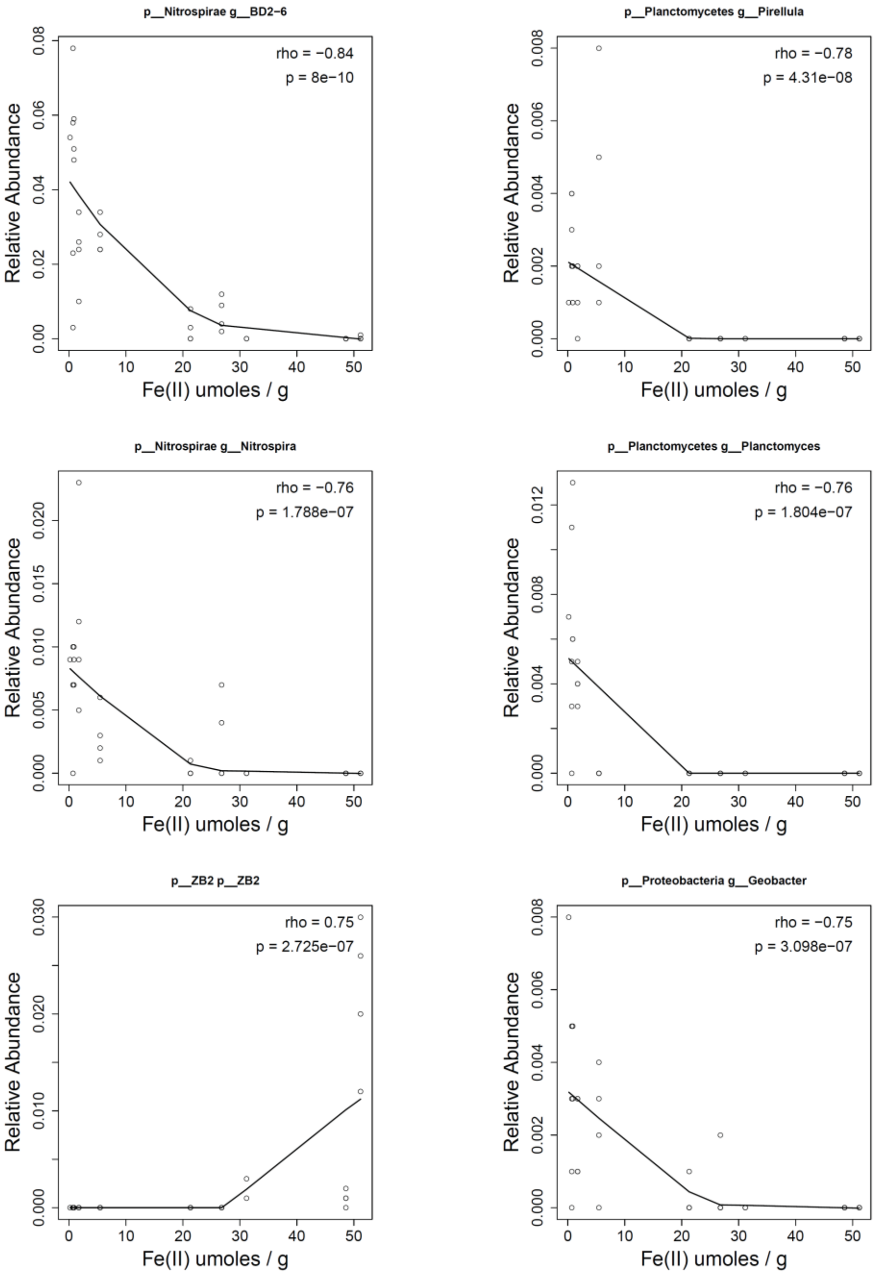

Figure S4 (cont).

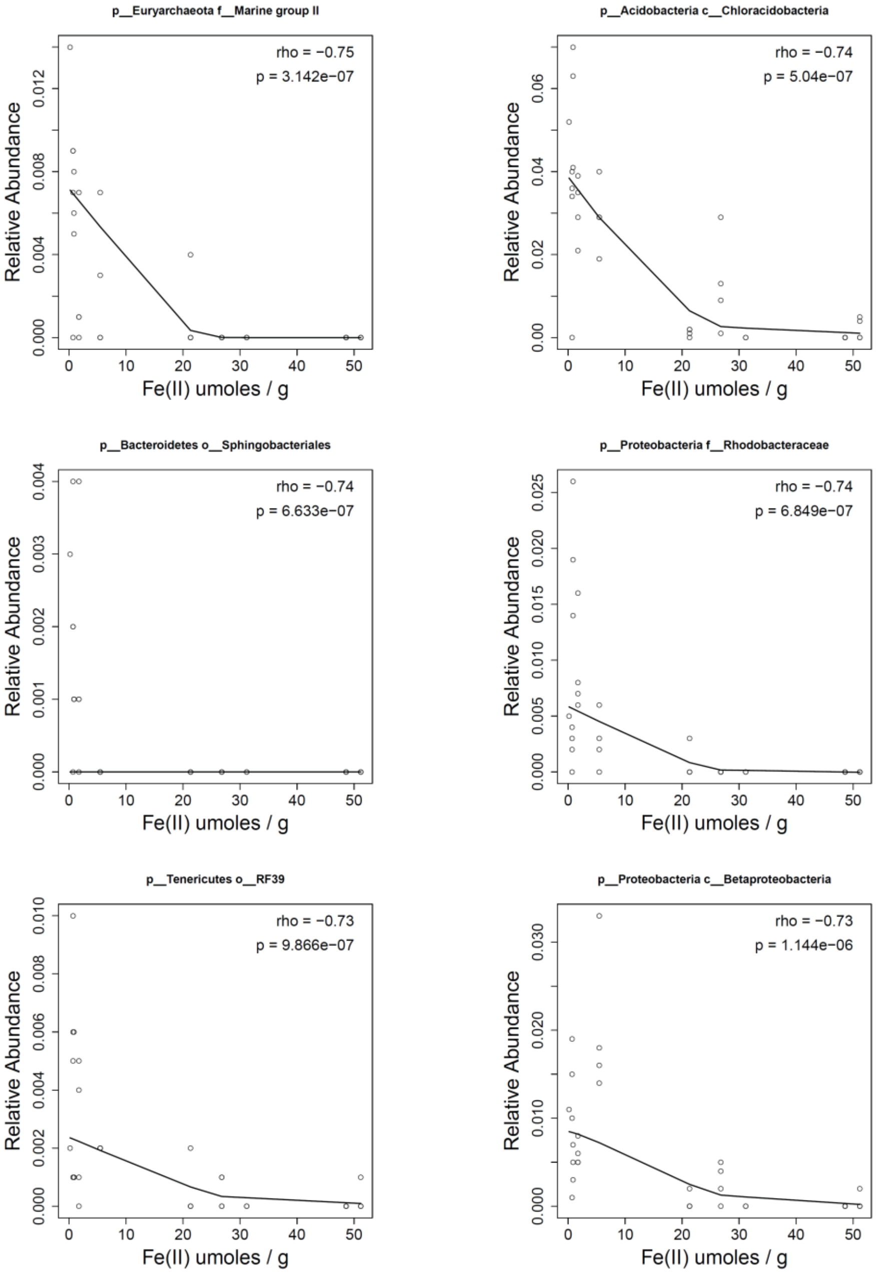

Figure S4 (cont).

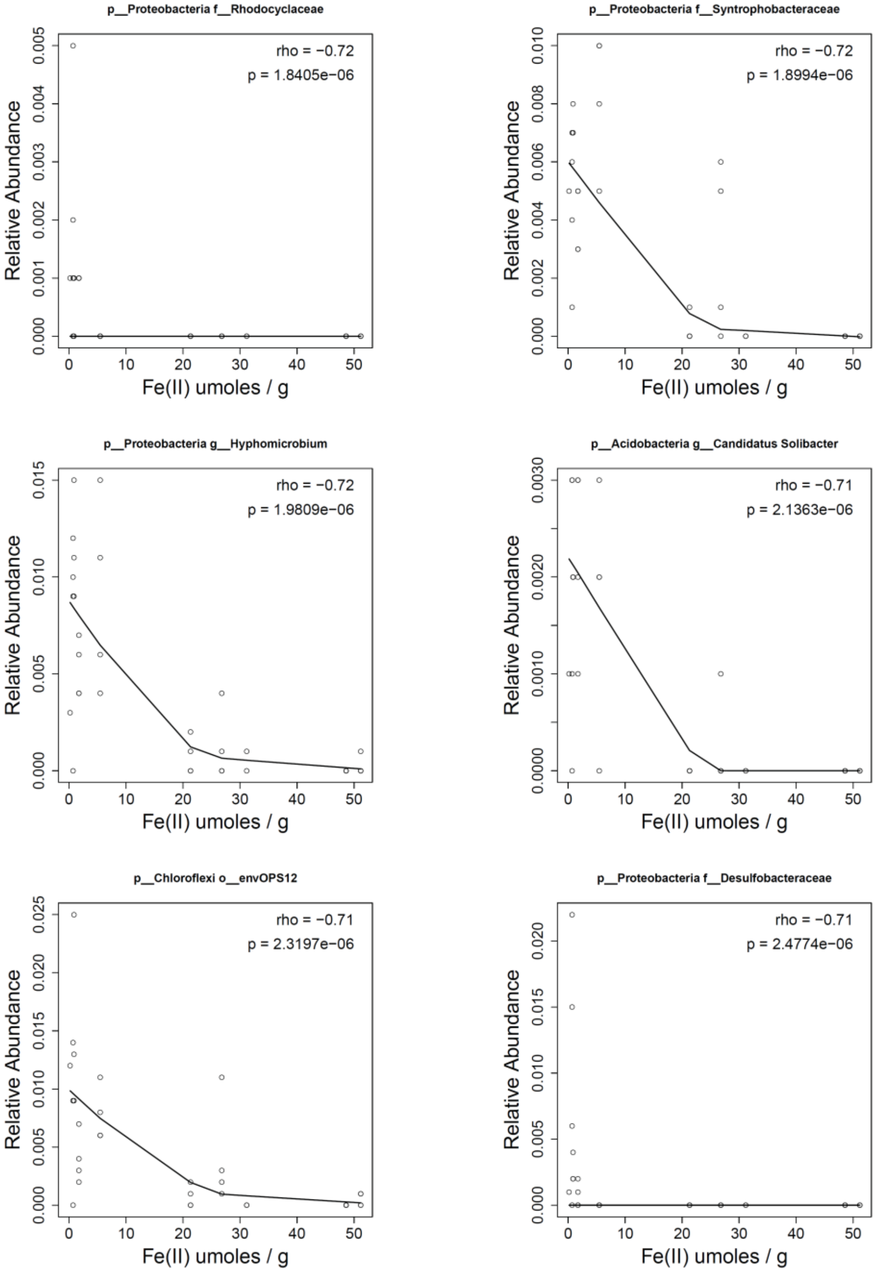

Figure S4 (cont).

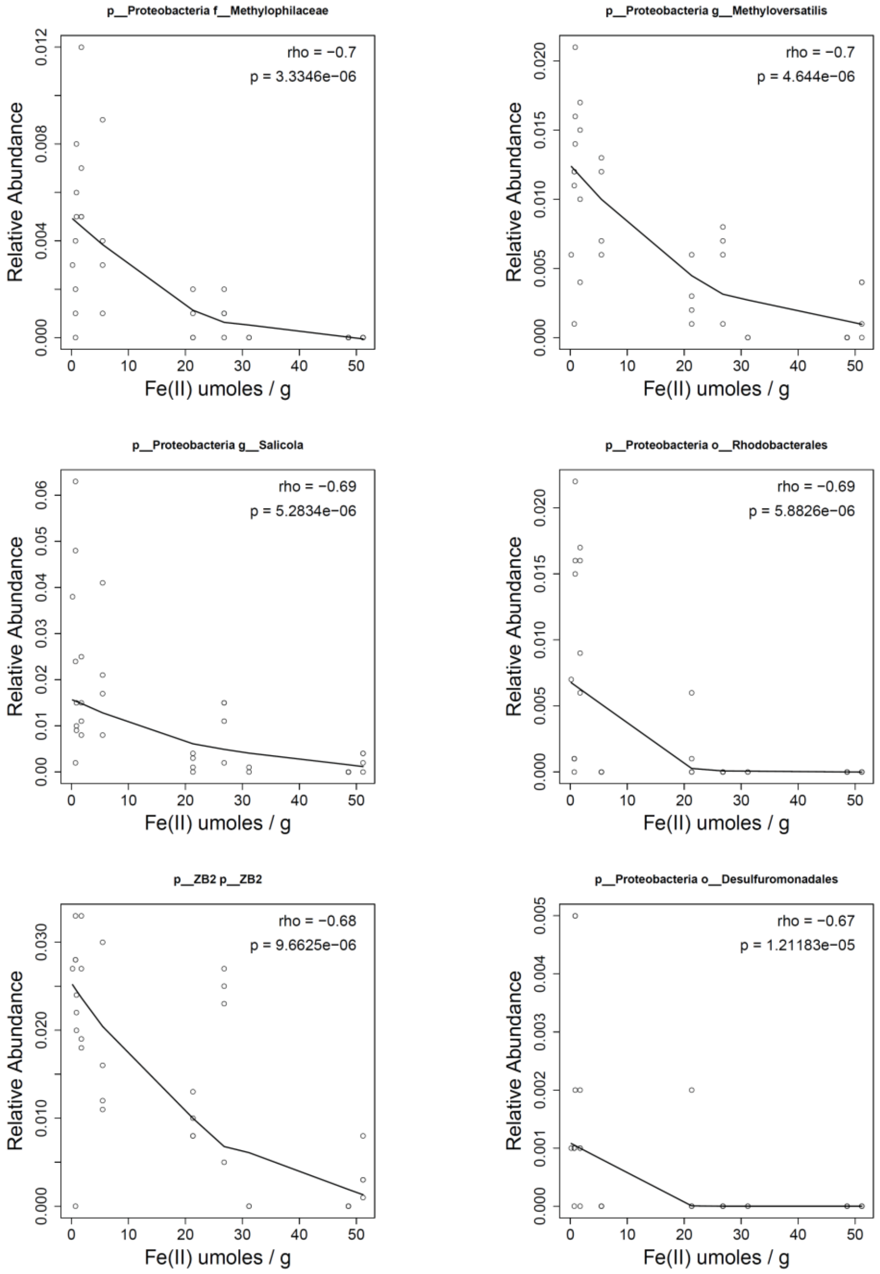

Figure S4 (cont).

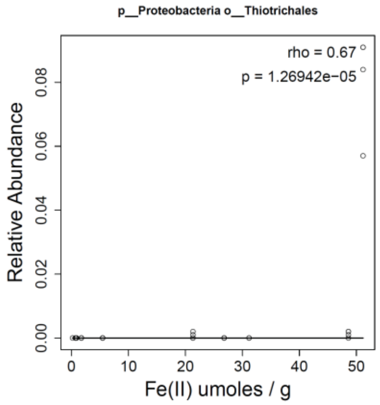

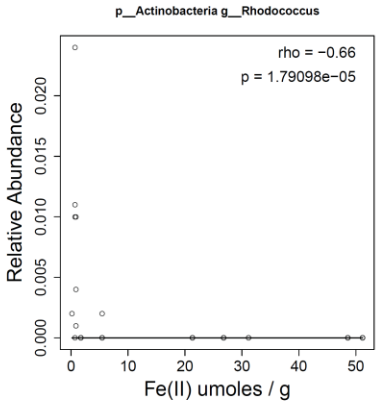

Figure S5. Vertical profiles—relative to the elevation of the transition reactive facies—of aqueous geochemical analytes from well C6199. Solid circles and 'x' symbols indicate values that were above or below the detection limit, respectively. The black and grey horizontal dashed lines indicate elevations of the Hanford-Ringold boundary and the transition reactive facies, respectively. The yellow box indicates the range of elevations (relative to the elevation of the transition reactive facies) for which microbial samples were analyzed from wells C7870 and C7867.

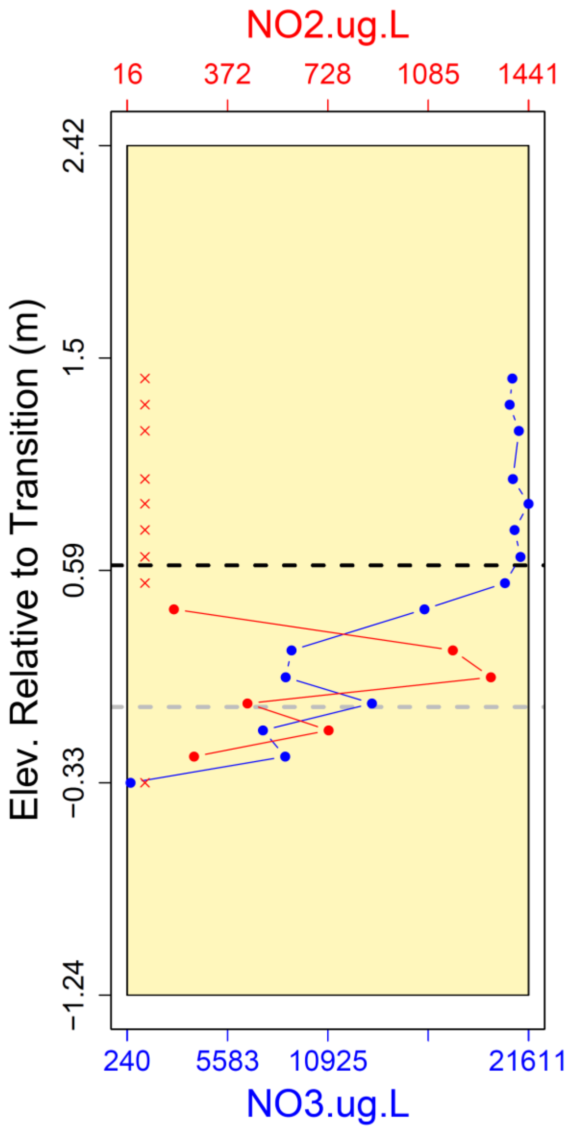

Supplement: Supplementary Information [file srep30553-s1.pdf]
